# Integrative Pan-Cancer Analysis Reveals Decreased Melatonergic Gene Expression in Carcinogenesis and *RORA* as a Prognostic Marker for Hepatocellular Carcinoma

**DOI:** 10.3389/fonc.2021.643983

**Published:** 2021-03-25

**Authors:** Yi Zou, Huaqin Sun, Yating Guo, Yidan Shi, Zhiyu Jiang, Jingxuan Huang, Li Li, Fengle Jiang, Zeman Lin, Junling Wu, Ruixiang Zhou, Yuncai Liu, Lu Ao

**Affiliations:** ^1^Department of Automation and Key Laboratory of China MOE for System Control and Information Processing, Shanghai Jiao Tong University, Shanghai, China; ^2^Department of Bioinformatics, Fujian Key Laboratory of Medical Bioinformatics, School of Basic Medical Sciences, Fujian Medical University, Fuzhou, China; ^3^Key Laboratory of Ministry of Education for Gastrointestinal Cancer, School of Basic Medical Sciences, Fujian Medical University, Fuzhou, China; ^4^Department of Cell Biology and Genetics, School of Basic Medical Sciences, Fujian Medical University, Fuzhou, China; ^5^Department of Human Anatomy, Histology and Embryology, School of Basic Medical Sciences, Fujian Medical University, Fuzhou, China

**Keywords:** melatonergic genes, pan-cancer analysis, expressional alteration, cancer occurrence and progression, prognosis

## Abstract

**Background:**

Melatonin has been shown to play a protective role in the development and progression of cancer. However, the relationship between alterations in the melatonergic microenvironment and cancer development has remained unclear.

**Methods:**

We performed a comprehensive investigation on 12 melatonergic genes and their relevance to cancer occurrence, progression and survival by integrating multi-omics data from microarray analysis and RNA sequencing across 11 cancer types. Specifically, the 12 melatonergic genes that we investigated, which reflect the melatonergic microenvironment, included three membrane receptor genes, three nuclear receptor genes, two intracellular receptor genes, one synthetic gene, and three metabolic genes.

**Results:**

Widely coherent underexpression of nuclear receptor genes, intracellular receptor genes, and metabolic genes was observed in cancerous samples from multiple cancer types compared to that in normal samples. Furthermore, genomic and/or epigenetic alterations partially contributed to these abnormal expression patterns in cancerous samples. Moreover, the majority of melatonergic genes had significant prognostic effects in predicting overall survival. Nevertheless, few corresponding alterations in expression were observed during cancer progression, and alterations in expression patterns varied greatly across cancer types. However, the association of melatonergic genes with one specific cancer type, hepatocellular carcinoma, identified *RORA* as a tumor suppressor and a prognostic marker for patients with hepatocellular carcinoma.

**Conclusions:**

Overall, our study revealed decreased melatonergic gene expression in various cancers, which may help to better elucidate the relationship between melatonin and cancer development. Taken together, our findings highlight the potential prognostic significance of melatonergic genes in various cancers.

## Introduction

Melatonin, a hormone produced mainly by the pineal gland, has many functions in regulating physiological processes, such as sleep, circadian rhythms, immunomodulation, anti-inflammatory signaling, and vasoconstriction (1). Melatonin exerts these actions *via* both receptor-dependent and -independent mechanisms. Melatonergic receptors include membrane-bound receptors, such as melatonin receptor type 1A (MT1, encoded by *MTNR1A*) and 1B (MT2, encoded by *MTNR1B*) (2, 3), and nuclear retinoic acid receptor-related orphan receptors (RORs; consisting of RORalpha, RORbeta and RORgamma) (4). Melatonin also binds to intracellular proteins, such as calmodulin 1 (CALM1), to modulate neurodevelopment (5). Additionally, N-Ribosyldihydronicotinamide: Quinone Reductase 2 (NQO2) has been identified as a melatonin-binding site (6) and G Protein-Coupled Receptor 50 (GPR50) has been reported to dimerize MT1 receptor to inhibit melatonergic signaling (7). Acetylserotonin O-methyltransferase (ASMT) is the final enzyme in the biosynthetic pathway of melatonin (8). In humans, melatonin is metabolized by hepatic cytochromes, including cytochrome P450 (CYP) 1 family members: CYP1A1, CYP1A2 and CYP1B1, to 6-hydroxymelatonin (9, 10). Importantly, melatonergic levels are restricted by both biosynthetic and metabolic processes, and expressional alterations in these melatonergic genes influence the melatonergic microenvironment (11).

In the last several decades, accumulating evidence has demonstrated that melatonin plays an important role in modulating pathological processes. Many studies have shown a protective role of melatonin in the occurrence and progression of cancer (12–14). For instance, low melatonergic levels have been reported to be associated with a high risk of breast cancer (15, 16). Moreover, melatonergic genes has a therapeutic perspective (17, 18). For example, overexpression of MT1 receptor was shown to enhance the growth suppressive effects of melatonin in breast cancer and melanoma cell lines (19, 20). Therefore, a comprehensive study investigating the relationship between alterations in the melatonergic microenvironment and cancer development, progression, and prognosis is needed.

In 2020, liver cancer ranks the fifth in cancer incidence and the third in tumor related mortality worldwide (21). Sorafenib is the first target drug approved for the treatment of hepatocellular carcinoma (HCC), a major type of liver cancer, which can prolong the overall survival time with a response rate of only 2% (22, 23). Our previous study has shown the anticancer property of melatonin in HCC (24) and increasing evidences have presented that melatonin can increase the sensitivity of HCC to sorafenib (25, 26). Thus, addressing melatonergic microenvironment in HCC is helpful to tailor individualized treatments for patients.

In the present study, we leveraged the large-scale gene expression profiles in the Gene Expression Omnibus (GEO) and RNA sequencing (RNA-seq) transcriptomic data in The Cancer Genome Atlas (TCGA) to characterize associations between genes reflecting the melatonergic microenvironment and cancer development across multiple cancer types. We comprehensively evaluated the expressional alterations of 12 melatonergic genes in 6,014 patients across 11 cancer types from GEO and TCGA databases. Genetic, epigenetic, and prognostic patterns of melatonergic genes were investigated by integrating multi-omic data from TCGA. Furthermore, the association of melatonergic genes with carcinogenesis was specifically investigated in HCC. Collectively, our findings may aid in better understanding the impacts of melatonin on cancer development, progression, and prognosis.

## Materials and Methods

### Data Sources and Data Preprocessing

All gene expression datasets of 6,014 cancerous samples and 1,211 matched normal samples for 11 cancer types analyzed in the present study are summarized in **Table 1**, and the cancer types included breast cancer (BRCA), colon and rectal adenocarcinoma (COAD), esophageal squamous cell carcinoma (ESCA), head and neck squamous cell carcinoma (HNSC), kidney clear cell carcinoma (KIRC), liver hepatocellular carcinoma (LIHC), lung adenocarcinoma (LUAD), lung squamous cell carcinoma (LUSC), pancreatic adenocarcinoma (PAAD), prostate adenocarcinoma (PRAD), and stomach adenocarcinoma (STAD). Each cancer type included the following two datasets: (1) microarray data from GEO; and (2) RNA-seq data with metastasis information from TCGA (27). The RNA-seq data for pancreatic cancer were from GEO due to an insufficient number of matched cancerous and normal samples in TCGA. Metastasis information for samples from TCGA is shown in **Supplementary Table S1**. For the microarray data, the raw mRNA expression data of cancerous and normal samples were downloaded and the robust multi-array average algorithm was used for background adjustment (28). For the RNA-seq data, gene expression values of cancerous and normal samples were represented by raw counts and limma was used to remove batch effects (29). Then, these data underwent TMM normalization (30).

**Table 1:** Description of datasets used in this study.

Cancer	Dataset	Type	Normal	Cancer			
				Exp	Methylation	Mutation	CNV
BRCA	TCGA	RNA-seq	113	1102	793	974	1080
	GSE10810	Microarray	27	31	–	–	–
COAD	TCGA	RNA-seq	41	476	313	367	451
	GSE21510	Microarray	25	123	–	–	–
ESCA	TCGA	RNA-seq	11	161	185	185	184
	GSE45670	Microarray	10	28	–	–	–
HNSC	TCGA	RNA-seq	44	500	528	511	522
	GSE6791	Microarray	14	42	–	–	–
KIRC	TCGA	RNA-seq	72	538	324	436	528
	GSE53757	Microarray	72	72	–	–	–
LIHC	TCGA	RNA-seq	50	371	377	373	370
	GSE14520	Microarray	220	225	–	–	–
LUAD	TCGA	RNA-seq	59	533	473	126	120
	GSE32863	Microarray	58	58	–	–	–
LUSC	TCGA	RNA-seq	49	502	370	533	501
	GSE18842	Microarray	45	31	–	–	–
PAAD	GSE119794	RNA-seq	10	10	–	–	–
	GSE16515	Microarray	16	36	–	–	–
PRAD	TCGA	RNA-seq	52	498	502	498	492
	GSE62872	Microarray	160	264	–	–	–
STAD	TCGA	RNA-seq	32	375	395	393	107
	GSE13911	Microarray	31	38	–	–	–
Total			1211	6014	4260	4396	4355

Multi-omic data for 10 of the 11 analyzed cancer types (i.e., all types except PAAD) were downloaded. Level-four somatic mutation data and copy number variation (CNV) data were obtained from TCGA *via* the Broad GDAC (http://gdac.broadinstitute.org/). Mutational data were identified by the CHASM algorithm (31) and only non−synonymous mutations were analyzed in the present study. A discrete copy-number-alteration status was given to each melatonergic gene in each sample by significant regions of gain or loss identified by the GISTIC 2.0 algorithm (*q* value<0.1) (32). Level-three DNA methylation data from the Illumina Infinium Human DNA methylation 450 platform were obtained from TCGA. For each CpG site, the methylation status was expressed as a beta-value in each sample, ranging from 0 to 1. We focused on the 264 CpG sites of 12 melatonergic genes, which were not “NA” in half of the above samples. The remaining “NA” values were replaced by the minimum beta value of this site in all samples.

The third independent LIHC dataset used in this study was the LIRI-JP dataset (n=231) from the International Cancer Genome Consortium (ICGC) database. Clinical information from the GSE14520, TCGA-LIHC, and LIRI-JP datasets are shown in **Supplementary Table S2**. LIHC microRNA (miRNA) expression profiles were downloaded from TCGA. The miRNAs regulating *RORA* were predicted from three commonly used databases, miRTarBase (http://miRTarBase.mbc.nctu.edu.tw/) (33), miRDB (http://mirdb.org/) (34), and Targetscan (http://www.targetscan.org/) (35).

### Identification of Differentially Expressed Melatonergic Genes Between Cancerous and Normal Tissue Samples

Student’s *t*-tests and edgeR were used to identify differentially expressed melatonergic genes between cancerous and normal samples *via* microarray analysis and RNA-seq, respectively (36, 37). The Benjamini and Hochberg (BH) procedure was used to control for the false discovery rate (FDR) (38). The differentially expressed statuses of melatonergic genes in cancerous samples were assigned to 1 for overexpression and -1 for underexpression for significant alterations compared to those in normal samples. The differentially expressed status was assigned to 0.5 or -0.5 in cancerous samples if the corresponding expression level was non-significantly greater than or less than that in normal samples. The final differentially expressed status of each melatonergic gene was calculated as follows:

$$S=\{\begin{array}{c} \sum^{}s_{array}+s_{seq},\;sgn\left(s_{array}\right)=sgn\left(s_{seq}\right)\\ \;\;\;\;\;\;\;\;0,\;\;\;\;\;\;\;\;\;sgn\left(s_{array}\right)\neq sgn\left(s_{seq}\right) \end{array}$$

*s_array_* and *s_seq_*, represent the differentially expressed statuses from the microarray dataset and RNA-seq dataset, respectively. For example, if a gene was significantly overexpressed in the RNA-seq data (FDR<0.05, fold change (FC)>0) and non-significantly overexpressed in the microarray data (FDR>0.05, *t*>0), then *s_array_* and *s_seq_* were 1 and 0.5, respectively, and its final differentially expressed status *S* was set as 1.5; if it was significantly overexpressed in the RNA-seq data (FDR<0.05, FC>1) but non-significantly underexpressed in the microarray data (FDR>0.05, *t*<0), then *s_array_* and *s_seq_* were 1 and -0.5, respectively, and its final differentially expressed status status *S* was set to 0. The FDR value below 0.05 was considered to be statistical significance. The differentially expressed statuses of each melatonergic gene in a particular cancer type were shown in **Supplementary Tables S3****,** **S4**.

### Statistical Analysis

The Wilcoxon rank sum test was used to identify differential methylation sites between cancerous and normal samples (39). If a gene was mapped by multiple CpG sites that were both hypermethylated and hypomethylated, the gene was not included in the following analysis. When all the CpG sites of a gene were differentially hypermethylated or hypomethylated, the gene was considered to be a differentially methylated gene. The univariate Cox proportional-hazards regression model was used to evaluate the correlation between gene expression levels and patient survival time. Student’s *t*-tests were used to identify differentially expressed melatonergic genes between metastatic samples with lymph node or distant metastasis (N+M+) and non-metastatic samples without lymph node or distant metastasis (N0M0). One-way analyses of variance (ANOVAs) and Tukey’s *post-hoc* tests were used to determine whether the mean expression values of melatonergic genes were significantly different among patients at different cancer stages. The multivariate Cox proportional-hazards regression model was used to evaluate the independent prognostic value of melatonergic genes after adjusting for clinical factors including serum alpha-fetoprotein (AFP), cancer size, and TNM stage (40). Patients were then dichotomized into high and low subgroups according to their median expression levels of melatonergic genes. The log-rank test (41) was used to evaluate whether survival was significantly different between these two subgroups, and the Kaplan-Meier method was used to represent the survival probability over time. Kaplan-Meier analysis and log-rank tests were also used to determine the association of somatic mutations (*TP53*/*CTNNB1*) in combination with the expression level of *RORA* in LIHC. All data analyses were performed using the R software package version 3.6.0.

### *RORA*-Related Functional Enrichment Analysis

Pearson correlation analysis was conducted to identify genes that were positively or negatively correlated with *RORA* expression in the corresponding datasets. Then, 223 pathways, excluding human disease pathways, were obtained from the KEGG database (https://www.kegg.jp/kegg/pathway.html). For each dataset, functional enrichment analysis was used to annotate biological pathways for *RORA*-positive-related and *RORA*-negative-related genes. The hypergeometric distribution model was used to determine significantly enriched pathways of these genes (42).

## Results

### Overview of Melatonergic Gene Expression in Cancer Development

**Figure 1:**
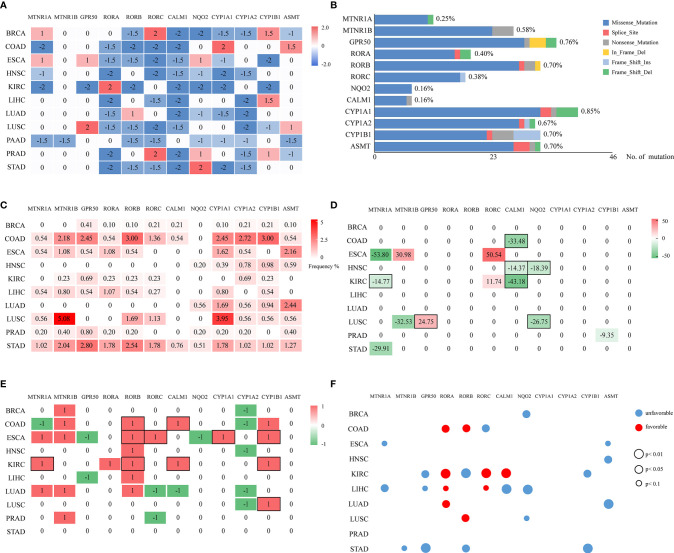
Multi-omic investigation and prognostic impacts of melatonergic genes across cancers. **(A)** Heatmap of the differentially expressed statuses of 12 melatonergic genes between cancerous and normal samples. The number on each cell represents its differentially expressed status. Red and blue indicate differential overexpression and underexpression, respectively. **(B)** Summary of mutated melatonergic genes. The x-axis denotes the total numbers of mutations of melatonergic genes in all RNA-seq samples across 11 cancer types. The length of the bar represents the proportion of mutated samples, which is marked on the right of the bar. **(C)** Mutational frequencies of melatonergic genes in certain cancer types. The numbers on the cells represent mutational percentages. **(D)** CNV frequencies of melatonergic genes across different cancer types. Numbers on the cells represent the CNV percentages, while red (greater than zero) and green (less than zero)represent amplification and deletion, respectively. **(E)** Heatmap of the differential-methylation statuses of melatonergic genes between cancerous and normal samples. Red (1) and green (-1) indicate hypermethylation and hypomethylation, respectively, in cancerous samples. The black boxes in **(D, E)** indicate that alterations in copy number or methylation are consistent with alterations in transcriptional expression. **(F)** Transcriptome-based prognostic impacts of melatonergic genes on OS across different cancer types. The univariate Cox proportional-hazards regression model was used to evaluate correlations. Red and blue denote favorable and unfavorable prognostic effects, respectively. The size of each circle indicates its significant *p*-value, whereas no circle denotes that there was no significant prognostic effect.

A total of 6,014 cancer samples of 11 cancer types were analyzed. A heatmap of the differentially expressed statuses of melatonergic genes between cancerous and normal samples is shown in **Figure 1A** and **Supplementary Tables S5**, **S6**. The majority of nuclear receptor genes, intracellular genes, and metabolic genes were underexpressed to varying degrees across cancer types, which is consistent with previous studies (43–45). Strikingly, *CALM1* was significantly underexpressed in all cancer types except for PAAD, whereas *CYP1A2* was underexpressed in eight cancer types (e.g., BRCA and LIHC). Additionally, some genes exhibited low expression in most cancer types but had high expression in one or two cancer types, which may have been associated with heterogeneity across these cancer types. *RORA* was significantly underexpressed in eight cancer types but was overexpressed in KIRC, whereas *CYP1A1* was underexpressed in seven cancer types but was overexpressed in COAD. Interestingly, *RORC* was significantly underexpressed in seven hormone-independent cancers (COAD, ESCA, HNSC, LIHC, LUSC, PAAD, and STAD) but was overexpressed in hormone-dependent cancers (e.g., BRCA and PRAD). In contrast, membrane receptor genes and synthetic genes were rarely differentially expressed across multiple cancer types, suggesting that the biological actions of melatonin in these cancer types might be independent of membrane-bound melatonergic receptors.

In two cancer types, HNSC and PAAD, all the differentially expressed melatonergic genes were consistently underexpressed. Meanwhile, all except for one of the differentially expressed melatonergic genes were underexpressed in KIRC, LIHC, LUAD, and STAD. Notably, the synthetic gene, *ASMT*, and metabolic gene, *CYP1A1*, were both highly expressed in COAD; notably, the gastro intestinal tract is an extra-pineal source of melatonin production during the daytime (46). Additionally, the metabolic gene, *CYP1B1*, was overexpressed in LIHC, possibly because the liver is the main site for metabolism of melatonin (1). However, synthetic genes and metabolic genes were not significantly differentially expressed in STAD. These heterogeneous expression patterns of melatonergic genes are likely due to cancer heterogeneity and are suggestive of cancer-type-specific functions of melatonergic genes. The comprehensively underexpressed nuclear receptor genes and intracellular genes across multiple cancer types are consistent with a protective role of melatonin in carcinogenesis. In contrast, there was no consistent relationship between membrane receptor genes and cancer incidence.

### Multi-Omics Analyses of Alterations in Transcription of Melatonergic Genes

According to TCGA somatic mutation profiles, mutations in melatonergic genes were rare, with a mean mutation rate of 0.68% (**Figure 1B**). The first- and second-highest mutation frequencies were 5.08% for *MTNR1B* and 3.95% for *CYP1A1*, respectively, in LUSC (**Figure 1C**). *CYP1A1* showed the highest mean mutation frequency across all cancer types (1.30%). The top-three mean mutation rates were observed in COAD (1.61%), STAD (1.53%), and LUSC (1.18%).

Compared with the relatively rare mutations, the CNV profiles showed that some melatonergic genes exhibited high alteration rates; for example, *CALM1* had deletion rates of 43.18%, 33.48%, and 14.37% in COAD, KIRC, and HNSC (**Figure 1D**), respectively, which were in line with alterations in expression. The highest alteration rate of deletion and amplification were both observed in ESCA, which were 53.80% for *MTNR1A* and 50.54% for *RORC*. The deletions of three genes were consistent with concomitant decreases in transcriptional levels (e.g., *MTNR1A* in KIRC; *NQO2* in HNSC and LUSC; *CALM1* in COAD, HNSC, and KIRC), while amplification of *GPR50* coincided with increased transcriptional levels in LUSC. However, *RORC* was amplified in two cancers, ESCA and KIRC, which was not coincident with transcriptional alterations.

Differentially methylated alterations of melatonergic genes varied across cancer types, as shown in **Figure 1E**. Compared with those in normal samples, *RORB*, *CALM1*, *CYP1A1*, and *CYP1B1* were significantly differentially hypermethylated in multiple cancer types, which was consistent with their transcriptional underexpression (e.g., *RORB* in COAD, ESCA, and KIRC; *CALM1* in COAD and KIRC; *CYP1A1* in ESCA; *CYP1B1* in ESCA, KIRC, and LUSC). However, *CYP1A2* was significantly differentially hypomethylated in five cancer types, which was inconsistent with its transcriptional alterations. Moreover, *MTNR1B*, which was rarely differentially expressed in pan-cancer transcriptional analysis, was significantly differentially hypermethylated in five cancer types, whereas frequent differentially underexpressed *RORA* did not exhibit consistent hypermethylation.

In summary, these results of multi-omics data suggest that genomic and epigenetic alterations partially contributed to the broad transcriptional changes of melatonergic genes across various cancer types.

### Relationship Between Melatonergic Gene Expression and Cancer Survival

Correlations among mRNA levels of melatonergic genes and patient overall survival (OS) in different TCGA cancer types are shown in **Figure 1F**. The results showed that most melatonergic genes had either favorable or unfavorable prognostic effects on patient OS. Among them, six melatonergic genes were significant unfavorable factors in cancers (e.g., *MTNR1A* in ESCA and LIHC; *GPR50* in KIRC, LIHC, and STAD; *NQO2* in BRCA, LIHC, and LUSC; *CYP1B1* in KIRC and STAD; *ASMT* in ESCA, HNSC, and LUAD; and *MTNR1B* in STAD). *RORA* was a significant favorable factor in COAD, KIRC, LIHC, and LUAD. Moreover, the effects of some melatonergic genes varied across cancer types; for example, *RORB* was associated with unfavorable survival for KIRC and STAD, while it was associated with favorable survival for COAD and LUSC. These results might be attributed to the complicated and variable mechanisms of different melatonergic genes in different cancers.

In addition, all favorable factors in COAD, LIHC, LUAD, and LUSC, as well as *CALM1* in KIRC, were also significantly differentially underexpressed in in the corresponding cancer types. Similarly, higher expression of *MTNR1A*, as an unfavorable factor, had a significantly shorter OS in ESCA. These results suggest that these melatonergic genes exert a coherent influence on the development and prognosis of various cancers.

### Relationship Between Melatonergic Gene Expression and Cancer Progression

**Figure 2:**
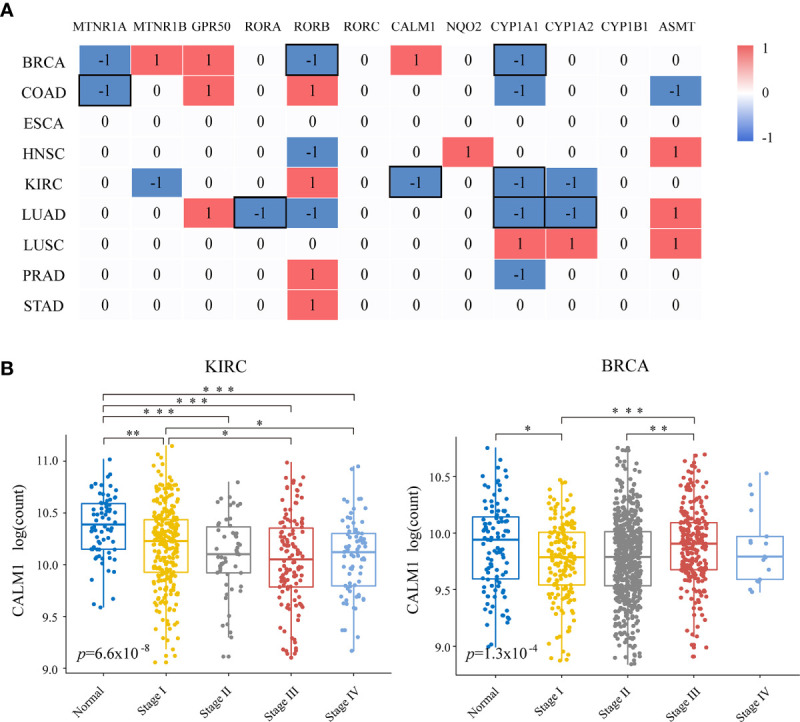
Relationships of melatonergic genes with cancer progression. **(A)** Differentially expressed melatonergic genes between metastatic samples with lymph node metastasis or distant metastasis (N+M+) and non-metastatic samples with neither lymph node metastasis nor distant metastasis (N0M0). Red (1) and blue (-1) indicate significant overexpression and underexpression, respectively. The black box represents the consistent differentially expressed patterns in cancer development and progression. **(B)** Expression levels of *CALM1* at different cancer stages in KIRC and BRCA. An ANOVA and Tukey’s *post-hoc* tests were used for pairwise comparisons. *, **, and *** indicate *p* < 0.05, *p* < 0.01, and *p* < 0.001, respectively.

Next, we examined the relationship between melatonergic gene expression and cancer progression. All cancerous samples from TCGA were classified into two subgroups: (1) metastatic samples with lymph node metastasis or distant metastasis (N+M+); and (2) non-metastatic samples with neither lymph node metastasis nor distant metastasis (N0M0). LIHC was excluded due to there only being eight samples in the metastatic group. In contrast to the widely coherent expressional alterations between cancerous and normal samples across different cancer types, few expressional alterations of melatonergic genes correlated to cancer progression (**Figure 2A**). Moreover, melatonergic genes with significantly different expressional alterations between metastatic and non-metastatic samples varied greatly across cancer types. Some melatonergic genes showed opposite alterations in expression patterns across different cancers. For example, as the cancer stage increased, *CALM1* expression decreased in KIRC, while it increased in BRCA (**Figure 2B**). Only *CYP1A1* was significantly underexpressed during cancer progression in five cancer types, which was consistent with alterations in the development of three cancer types, namely, BRCA, KIRC, and LUAD. Meanwhile, consistent with the expressional alterations between cancerous and normal samples, significantly lower levels of some melatonergic genes were observed between the metastatic and non-metastatic groups (e.g., *MTNR1A* in COAD; *RORA* in LUAD; *RORB* in BRCA; *CALM1* in KIRC; *CYP1A2* in LUAD). In contrast, some melatonergic genes were significantly overexpressed during cancer progression and were opposite to their differentially expressed status between cancerous and normal samples (e.g., *RORB* in COAD, KIRC, and STAD; *CALM1* in BRCA; *CYP1A2* in LUSC). Strikingly, *GPR50* had the highest expression level in the metastatic group compared to that in the non-metastatic group in three cancer types (e.g., BRCA, COAD, and LUAD). Interestingly, higher levels of *ASMT* and *CYP1A1* were observed in COAD than in normal samples, whereas lower levels were observed in metastatic COAD than in non-metastatic COAD.

### Evaluation of Melatonergic Gene Expression in LIHC

**Figure 3:**
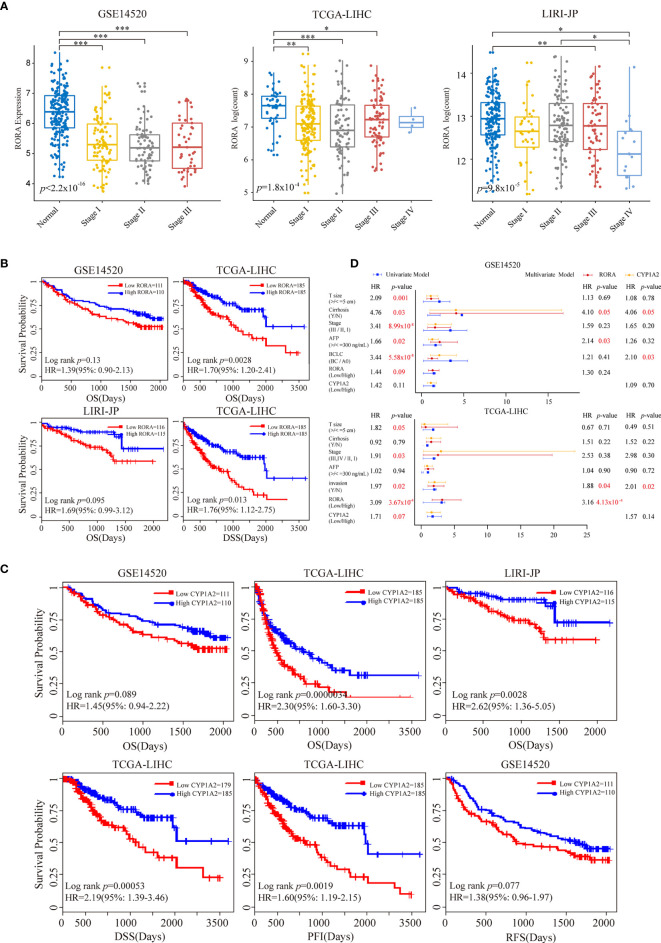
Prognostic impacts of *RORA* and *CYP1A2* in three independent LIHC datasets. Patients were grouped according to the median expression levels of *RORA* and *CYP1A2*. **(A)** Expression levels of *RORA* at different cancer stages of three datasets. An ANOVA and Tukey’s *post-hoc* tests were used for pairwise comparisons. *, **, and *** indicate *p* < 0.05, *p* < 0.01, and *p* < 0.001, respectively. Kaplan-Meier survival curves of *RORA* **(B)** and *CYP1A2* **(C)** on patient survival. **(D)** Univariate and multivariate Cox proportional hazard regression analysis of *RORA* and *CYP1A2*.

We further evaluated the relationship between melatonergic gene expression and LIHC in the third independent dataset, LIRI-JP. With a 5% FDR, the expression levels of *RORA*, *CALM1*, and *CYP1A2* were significantly lower in LIHC samples than in normal samples in all three datasets. *RORA* expression showed a decreased trend as the cancer AJCC stage increased (**Figure 3A**). Moreover, significant differences were observed between stage III–IV and stage I–II: TCGA-LIHC (*p*=0.0036) and LIRI-JP (*p*=0.0084). Then, we examined whether the expressional alterations of *RORA*, *CALM1*, and *CYP1A2* influenced the survival rates of LIHC. All cancer samples were classified into two subgroups according to the median expression levels of the three genes (high vs low expression) in the three independent datasets. The results showed that *RORA* and *CYP1A2* had favorable prognostic effects on OS, while *CALM1* had no significant influence. A higher expression of *RORA* was associated with a favorable OS in the three datasets and better disease-specific survival (DSS) in the TCGA-LIHC dataset (**Figure 3B**). Similarly, patients with a higher expression of *CYP1A2* had significantly better OS in the three datasets, better recurrence free survival (RFS) in the GSE14520 dataset, and better DSS and progression-free interval (PFI) in the TCGA-LIHC dataset (**Figure 3C**). Multivariate Cox regression analysis revealed that *RORA* was an independent prognostic factor in the TCGA-LIHC dataset after controlling for AFP, cancer size, and TNM stage (**Figure 3D**). Moreover, according to the Human Protein Atlas (HPA, https://www.proteinatlas.org/) database (47), the antibody staining proportions of protein RORA in HCC tissues were less than that in normal tissues. The detail information was shown in **Supplementary Figure S1**. The result suggested that the level of protein RORA might be underexpressed in HCC tissues.

**Figure 4:**
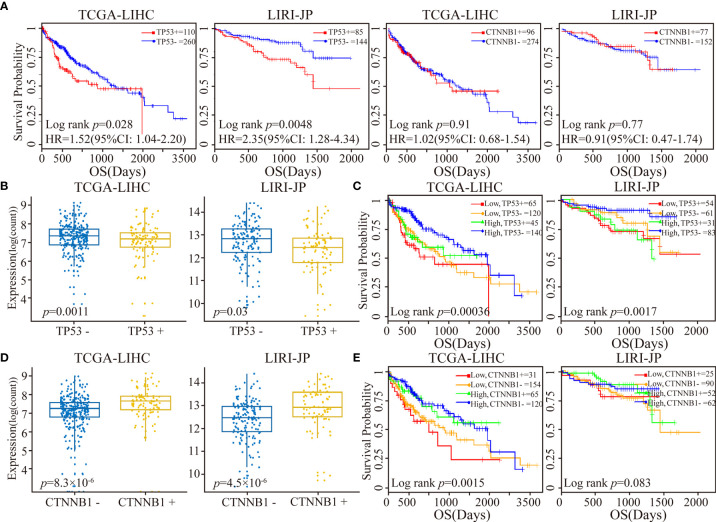
Patient stratification *via* the median expression level of *RORA* and *TP53*/*CTNNB1* mutational status. **(A)** Kaplan-Meier survival curves of *TP53* or *CTNNB1* mutations on OS. **(B)** Box plots of *RORA* expression levels in patients classified with *TP53* mutations. **(C)** Kaplan-Meier plots depicting a combined relation of *TP53* mutational status and *RORA* expression with OS in HCC patients. **(D)** Box plots of *RORA* expression levels in patients classified with *CTNNB1* mutations. **(E)** Kaplan-Meier plots depicting a combined relation of *CTNNB1* mutation status and *RORA* expression levels with OS in HCC patients.

In the TCGA-LIHC and LIRI-JP datasets with available mutation data, we next investigated the relationship between *RORA* expression and the mutation status of*TP53* and *CTNNB1*, two of the most commonly mutated genes in LIHC (48). Studies have consistently reported a strong association between *TP53* mutations and a poor prognosis, while results on the association of *CTNNB1* mutations with prognosis have been contradictory (49, 50). In the present study, we observed that *TP53* mutations were significantly associated with a worse OS, while there was no significant difference between *CTNNB1* mutations and OS (**Figure 4A**). Next, all cancer samples were classified into a mutant group and a wild-type group according to *TP53* or *CTNNB1* mutations. We observed that *RORA* exhibited a significantly lower expression level in the *TP53* mutant group than in the *TP53* wild-type group (**Figure 4B**). All samples were classified into four subgroups according to *TP53* mutations and the expression level of *RORA*. A significant difference in OS was observed in the four subgroups; patients harboring *TP53* mutations and low expression of *RORA* had the worst OS (**Figure 4C**). In the two *TP53* wild-type subgroups, a low expression level of *RORA* was significantly associated with a sharp decline in OS in the TCGA-LIHC cohort (*p*=0.0074) but had no significant association with OS in the LIRI-JP cohort (*p*=0.10). Furthermore, in the two subgroups with *TP53* mutations, the expression level of *RORA* was not associated with patient OS. Similar analyses were performed in the four subgroups according to *CTNNB1* mutations and the expression level of *RORA*. *RORA* exhibited a higher expression level in the *CTNNB1* mutant group than in the *CTNNB1* wild-type group (**Figure 4D**), while patients harboring *CTNNB1* mutations and low expression of *RORA* had the worst OS (**Figure 4E**). In the TCGA-LIHC dataset, the association of *RORA* expression and patient OS in the two subgroups with wild-type *CTNNB1* was significant (*p*=0.016), as was that of the two subgroups with *CTNNB1* mutations (*p*=0.034). Collectively, these results suggest that the *RORA* expression may be a prognostic marker for HCC patients.

**Figure 5:**
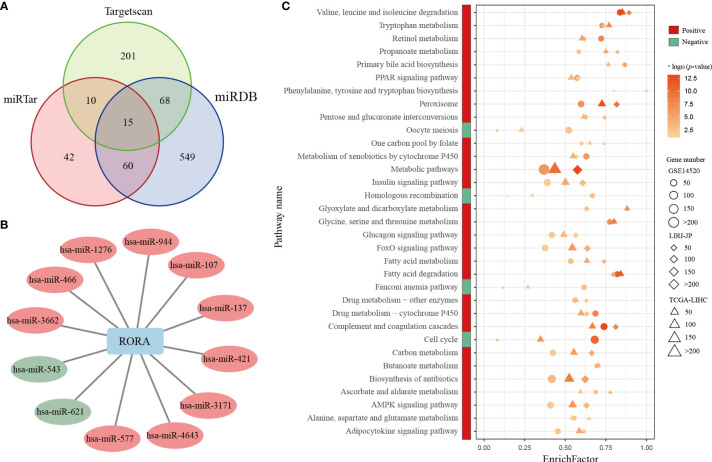
Differentially expressed miRNAs and common KEGG pathways enriched in *RORA*-related genes. **(A)** Venn diagram of miRNAs whichcan regulate *RORA* in the miRTarBase, miRDB, and Targetscan databases. **(B)** The interactions of differentially expressed miRNAs and *RORA*. Red and green circles indicate significant overexpression and underexpression miRNA in LIHC, respectively (Student’s *t*-test, FDR<0.05). **(C)** Red and green boxes indicate pathways enriched in *RORA*-positively regulated and *RORA*-negatively related genes, respectively. The three shapes indicate the pathways enriched in different datasets: circles for GSE14520, diamonds for LIRI-JP, and triangles for TCGA-LIHC. The sizes of shapes indicate the numbers of *RORA*-related genes enriched in a specific pathway. The *p* values of KEGG pathways were adjusted by BH (FDR < 5%), and -log10(*p*) was used to generate the heatmap.

Because *RORA* showed a low mutation frequency in LIHC and few genomic or epigenetic alternation, then we analyzed the expressions of miRNAs which could regulate the expression of *RORA*. *RORA* has been predicted to have miRNA-binding sites, e.g., hsa-miR-3662 and hsa-miR-4643 (51–54). A total of 153 miRNAs which were predicted or verified to target *RORA* in at least two databases of miRTarBase, miRDB, and Targetscan were selected (**Figure 5A**). 12 miRNAs were differentially expressed (Student’s *t*-test, FDR<0.05, **Figure 5B**), of which 10 miRNAs were overexpressed in cancerous samples compared with normal samples. It has been reported that hsa-miR-137 could repress *RORA* in autism spectrum disorders (55). The result suggested that the abnormal expression pattern of *RORA* might be attributed to the dysregulation of miRNA.

Pearson correlation analyses identified 3,633, 6,909, and 8,538 genes that were significantly positively correlated with *RORA* expression in the GSE14520, TCGA-LIHC, and LIRI-JP datasets (*p*<0.05), respectively. With a 5% FDR, these genes were significantly enriched in 29 common biological pathways in all three datasets (**Figure 5C**). These pathways were mostly associated with metabolic mechanisms related to the development of HCC, including fatty acid metabolism and xenobiotic metabolism *via* cytochrome P450. Likewise, 4,859, 3,343, and 449 genes were significantly negatively correlated with *RORA* expression in the GSE14520, TCGA-LIHC, and LIRI-JP datasets, respectively, which were enriched in four common biological pathways (FDR<0.05), namely, those involving homologous recombination, Fanconi anemia, cell-cycle progression, and oocyte meiosis. Collectively, these results indicate that *RORA* might regulate these pathways to exhibit its favorable prognostic effects in HCC.

## Discussion

In the present study, we systematically investigated the relationships between melatonergic genes and the occurrence, progression, and prognosis of cancer across 11 cancer types. The results showed that nuclear receptor genes, intracellular receptor genes, and metabolic genes were coherently underexpressed in multiple cancer types, whereas membrane receptor genes and synthetic genes were rarely differentially expressed. Multi-omic analyses showed that the abnormal expression patterns of melatonergic genes were partially attributed to genomic and epigenetic alterations. Furthermore, the majority of melatonergic genes had significant prognostic effects in various cancers. Nevertheless, few alterations in melatonergic gene expression were observed during cancer progression.

Recently, Lv et al. performed a genome-wide characterization of the melatonergic microenvironment in human cancers using TCGA RNA-seq data and revealed that the rates of melatonergic synthesis and metabolism were negatively correlated with mutational burden and clinical outcome (56). This previous study provided a key insight into the role of the melatonergic synthesis/metabolism system in cancer biology. However, this previous study only included one synthetic gene, *ASMT*, and three metabolic genes (*CYP1A1*, *CYP1A2*, and *CYP1B1*), and receptor genes were not assessed. Moreover, this previous study only analyzed TCGA RNA-seq data and did not included any microarray data. Notably, in the present study, we included both RNA-seq from TCGA and microarray data from GEO to analyze 12 melatonergic genes across 11 cancer types. For a particular cancer type, only melatonergic genes with a consistent differentially expressed status in both the RNA-seq and microarray datasets were assessed for transcriptional alterations. This approach may have helped to mitigate uncertainty in different platforms due to different design principles, and may have helped to yield more reproducible results.

Our investigation of melatonergic genes in three independent datasets identified *RORA* as a cancer suppresser and prognostic marker in LIHC, consistent with some previous studies (57–59). However, these previous studies were performed *in vitro* and/or identified changes in mRNA levels by quantitative reverse-transcriptase polymerase chain reaction. Our present study demonstrated a suppressive role of *RORA* in LIHC *via* high-throughput gene expression profiles in large-scale independent samples, revealing a relationship between *RORA* expression levels and clinicopathological features of LIHC. Furthermore, we found that *RORA* was significantly underexpressed in eight cancer types but was overexpressed in KIRC, suggesting that the biological impacts of expressional alterations in melatonergic genes are dependent on the cancer type. A study has presented that *RORA* is involved in kidney development (60). The overexpression of *RORA* in KIRC might be due to its tissue specificity. Such cancer-type specificities revealed *via* pan-cancer analyses suggest that melatonergic genes may have clinical utility.

Many studies have reported anticancer effects of melatonin through induction of apoptosis and cell-cycle arrest (13, 54, 61). The results of our functional enrichment analysis of *RORA*-negatively correlated genes in LIHC were consistent with findings from previous studies, such as the finding of an enrichment in cell-cycle pathways. The coincidence of underexpression of *RORA* and overexpression of *RORA*-negatively correlated genes might promote cell-cycle progression of cancer cells, which would further promote cancer development. *RORA*-positively correlated genes in LIHC were mostly enriched in metabolism-related pathways, which might be due to the liver being the main metabolic organ disrupted in LIHC.

In our study, *CYP1A2* was significantly differentially hypomethylated in five types of cancer, which was significantly differentially underexpressed in four of them. The inconsistence between hypomethylation and underexpression of gene is possibly due to that hypomethylation always allies with gene activation to enhance gene expression (62). Moreover, DNA hypomethylation in some regions may further leads to gene silence (63). The metabolic genes, *CYP1A1* and *CYP1A2*, were widely underexpressed in multiple cancer types, whereas *CYP1B1* exhibited different alterations in expression patterns. This result may be due to *CYP1B1* having a ubiquitous extra hepatic distribution and being primarily responsible for the metabolism of melatonin in the brain (10). Furthermore, relevant pathways of melatonergic genes involved in different types of cancers are worthy of further investigation in future studies. Melatonin has been reported to play a protective role in cancer metastasis (64). However, our present findings revealed that a minority of melatonergic genes were differentially expressed between metastatic and non-metastatic samples, and that alterations in expression patterns varied greatly across cancer types. Therefore, the protective effects of melatonin during cancer progression require further cancer-type-specific analysis.

## Data Availability Statement

The original contributions presented in the study are included in the article/**Supplementary Material**. Further inquiries can be directed to the corresponding authors.

## Author Contributions

All authors had full access to all the data in the study and take responsibility for the integrity of the data and the accuracy of the data analysis. LA and RZ conceived and supervised the study. YZ, HS, and YG acquired the data and participated in the statistical analysis. ZJ and YS participated in the statistical analysis and drew the figures. JH, LL, FJ, ZL, and JW interpreted the results. YZ, HS, and YG drafted the manuscript. LA and YL revised the manuscript. All authors contributed to the article and approved the submitted version.

## Funding

This work was supported by the National Natural Science Foundation of China (Grant No. 81602738), the Joint Scientific and Technology Innovation Fund of Fujian Province (Grant No. 2018Y9065), the Joint research program of health and education in Fujian Province (Grant No. 2019-WJ-32), the Natural Science Foundation of Fujian Province (Grant No. 2020J01600), and Undergraduate Training Programs for Innovation and Entrepreneurship of Fujian Medical University (Grant No. C20092).

## Conflict of Interest

The authors declare that the research was conducted in the absence of any commercial or financial relationships that could be construed as a potential conflict of interest.

## Supplementary Material

Supplementary Figure 1 IHC-based antibody-specific staining in HCC and normal liver tissues.

Supplementary Table 1 Metastasis information of TCGA samples.

Supplementary Table 2 Clinical characteristics of HCC patients in three datasets.

Supplementary Table 3 Score of differentially expressed status from microarray dataset, *s_array_*.

Supplementary Table 4 Score of differentially expressed status from RNA-seq dataset, *s_seq_*.

Supplementary Table 5 Differentially expressed status of melatonergic genes in the TCGA data.

Supplementary Table 6 Differentially expressed status of melatonergic genes in the microarray data.
