# Supplementary figures and images for: Integrative Pan-Cancer Analysis Reveals Decreased Melatonergic Gene Expression in Carcinogenesis and RORA as a Prognostic Marker for Hepatocellular Carcinoma
Source: Front Oncol. 2021 Mar 25;11:643983. doi: 10.3389/fonc.2021.643983 (PMC8029983; doi:10.3389/fonc.2021.643983)

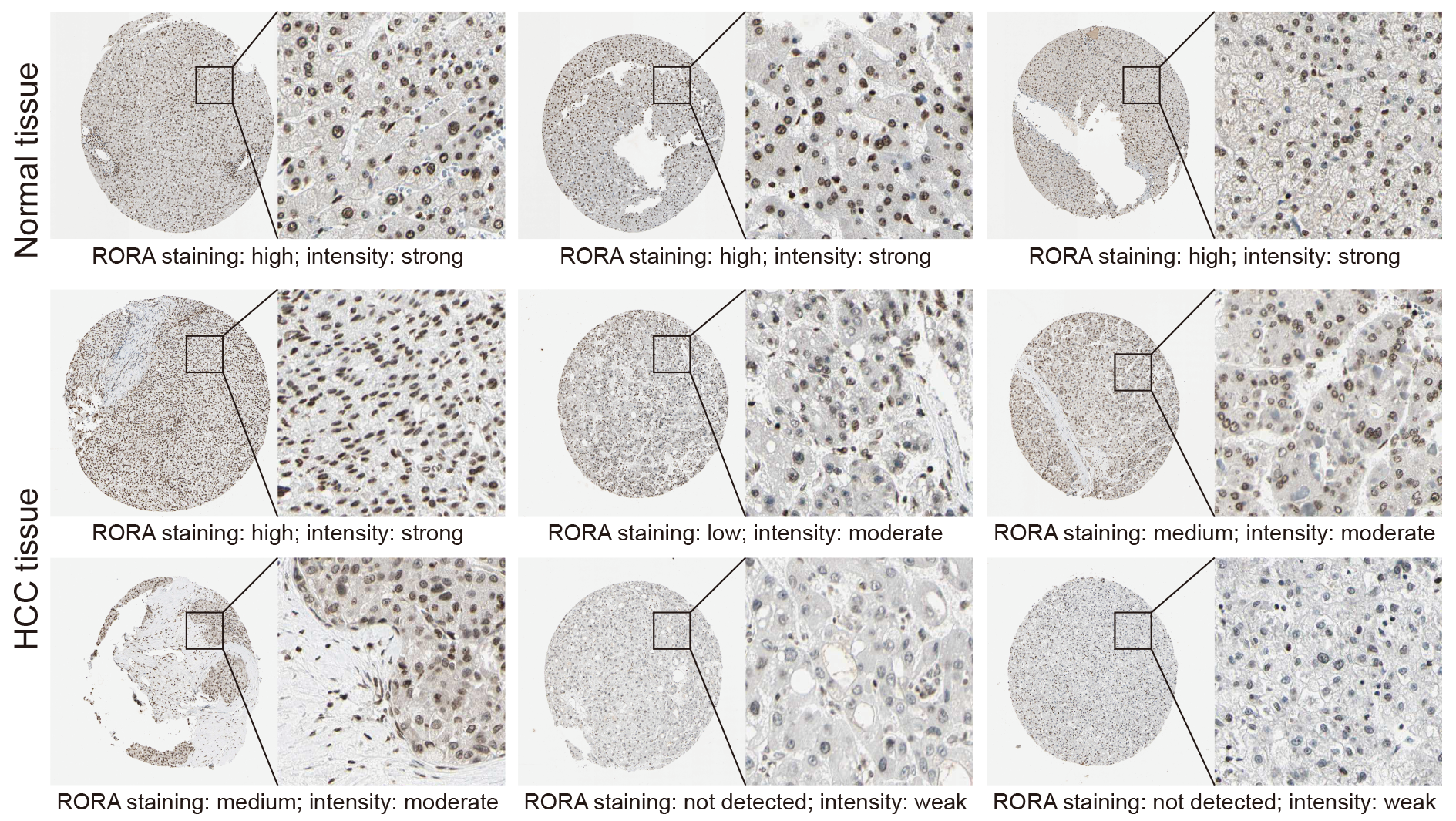

Supplement: Supplementary Figure 1 — IHC-based antibody-specific staining in HCC and normal liver tissues. [file Image_1.tif]
