# Supplementary material for: Integrative Pan-Cancer Analysis Reveals Decreased Melatonergic Gene Expression in Carcinogenesis and RORA as a Prognostic Marker for Hepatocellular Carcinoma
Source: Front Oncol. 2021 Mar 25;11:643983. doi: 10.3389/fonc.2021.643983 (PMC8029983; doi:10.3389/fonc.2021.643983)
Supplement: Supplementary Table 1 — Metastasis information of TCGA samples. [file Table_1.docx]

| Supplementary Table S1. Metastasis information of TCGA samples | | |
| --- | --- | --- |
| Cancer | N+M+ | N0M0 |
| BRCA | 564 | 458 |
| COAD | 202 | 242 |
| ESCA | 89 | 54 |
| HNSC | 235 | 80 |
| KIRC | 89 | 201 |
| LIHC | 8 | 228 |
| LUAD | 185 | 231 |
| LUSC | 180 | 259 |
| PAAD | 125 | 20 |
| PRAD | 68 | 225 |
| STAD | 248 | 103 |
