# Supplementary material for: Integrative Pan-Cancer Analysis Reveals Decreased Melatonergic Gene Expression in Carcinogenesis and RORA as a Prognostic Marker for Hepatocellular Carcinoma
Source: Front Oncol. 2021 Mar 25;11:643983. doi: 10.3389/fonc.2021.643983 (PMC8029983; doi:10.3389/fonc.2021.643983)
Supplement: Supplementary Table 2 — Clinical characteristics of HCC patients in three datasets. [file Table_2.docx]

| Supplementary Table S2. Clinical characteristics of HCC patients in three datasets | | | | |
| --- | --- | --- | --- | --- |
|  |  | **GSE14520** | **TCGA-LIHC** | **LIRI-JP** |
| Patient number | Total | 225 | 371 | 231 |
| Viral etiology | Hepatitis B | 225 (100%) | 97 (26%) |  |
|  | Hepatitis C |  | 49 (13%) |  |
|  | Hepatitis B & C |  | 6 (2%) |  |
|  | Negative |  | 200(54%) |  |
|  | Not available |  | 19(5%) | 231(100%) |
| Tumour size | Small (< =5cm) | 140 (62%) |  |  |
|  | Large (> 5cm) | 80 (36%) |  |  |
|  | Not available | 5 (2%) | 371 (100%) |  |
| Cirrhosis | Y | 203(90%) | 79(21%) |  |
|  | N | 18(8%) | 133(36%) |  |
|  | Not available | 4(2%) | 159(43%) | 231(100%) |
| AFP | Low (<= 300ng/mL) | 118 (52%) | 161 (59%) |  |
|  | High (> 300ng/mL) | 100 (44%) | 117(18%) |  |
|  | Not available | 7 (3%) | 93 (23%) | 231(100%) |
| BCLC stage | 0 | 20 (9%) |  |  |
|  | A | 148 (66%) |  |  |
|  | B | 22 (10%) |  |  |
|  | C | 29 (13%) |  |  |
|  | Not available | 6 (3%) | 371 (100%) | 231(100%) |
| TNM stage | I | 93 (41%) | 171 (46%) | 36(16%) |
|  | II | 77 (34%) | 86 (23%) | 106(29%) |
|  | III | 49 (22%) | 85 (23%) | 71(19%) |
|  | IV |  | 5 (1%) | 19(5%) |
|  | Not available | 6 (3%) | 24 (6%) |  |
| Invasion | Y |  | 109(29%) |  |
|  | N |  | 206(54%) |  |
|  | Not available | 225 (100%) | 56(15%) | 231(100%) |
