# Supplementary material for: Integrative Pan-Cancer Analysis Reveals Decreased Melatonergic Gene Expression in Carcinogenesis and RORA as a Prognostic Marker for Hepatocellular Carcinoma
Source: Front Oncol. 2021 Mar 25;11:643983. doi: 10.3389/fonc.2021.643983 (PMC8029983; doi:10.3389/fonc.2021.643983)
Supplement: Supplementary Table 3 — Score of differentially expressed status from microarray dataset, sarray. [file Table_3.docx]

| **Supplementary Table S3. Score of differentially expressed status from RNA-seq dataset,** $s_{\mathrm{seq}}$ $\boldsymbol{s}_{\boldsymbol{seq}}$ | | | |
| --- | --- | --- | --- |
| FDR | edgeR | $s_{\mathrm{seq}}$ $s_{seq}$ | $sgn(s_{seq})$ |
| <0.05 | FC>1 | 1 | 1 |
| >=0.05 | FC>1 | 0.5 | 1 |
| <0.05 | FC<1 | -1 | -1 |
| >=0.05 | FC<1 | -0.5 | -1 |
