# Supplementary material for: Integrative Pan-Cancer Analysis Reveals Decreased Melatonergic Gene Expression in Carcinogenesis and RORA as a Prognostic Marker for Hepatocellular Carcinoma
Source: Front Oncol. 2021 Mar 25;11:643983. doi: 10.3389/fonc.2021.643983 (PMC8029983; doi:10.3389/fonc.2021.643983)
Supplement: Supplementary Table 4 — Score of differentially expressed status from RNA-seq dataset, sseq. [file Table_4.docx]

| **Supplementary Table S4. Score of differentially expressed status from microarray dataset,** $\boldsymbol{s}_{\boldsymbol{array}}$ | | | |
| --- | --- | --- | --- |
| FDR | Student's *t*-tests | $s_{array}$ | $sgn(s_{array})$ |
| <0.05 | *t*>0 | 1 | 1 |
| >=0.05 | *t*>0 | 0.5 | 1 |
| <0.05 | *t*<0 | -1 | -1 |
| >=0.05 | *t*<0 | -0.5 | -1 |
