# Supplementary material for: Integrative Pan-Cancer Analysis Reveals Decreased Melatonergic Gene Expression in Carcinogenesis and RORA as a Prognostic Marker for Hepatocellular Carcinoma
Source: Front Oncol. 2021 Mar 25;11:643983. doi: 10.3389/fonc.2021.643983 (PMC8029983; doi:10.3389/fonc.2021.643983)
Supplement: Supplementary Table 5 — Differentially expressed status of melatonergic genes in the TCGA data. [file Table_5.docx]

| Supplementary Table S5. Differentially expressed status of melatonergic genes in the TCGA data | | | | | | | | | | | | |
| --- | --- | --- | --- | --- | --- | --- | --- | --- | --- | --- | --- | --- |
|  | MTNR1A | MTNR1B | GPR50 | RORA | RORB | RORC | CALM1 | NQO2 | CYP1A1 | CYP1A2 | CYP1B1 | ASMT |
| BRCA | 0.5 | 1 | -1 | -1 | -1 | 1 | -1 | -1 | -1 | -1 | 1 | -0.5 |
| COAD | -1 | 0.5 | 1 | -0.5 | -1 | -1 | -1 | 1 | 0.5 | 0.5 | 0.5 | 0.5 |
| ESCA | 0.5 | 0.5 | 0.5 | -0.5 | -0.5 | -1 | -0.5 | 0.5 | -0.5 | -1 | -1 | -1 |
| HNSC | -0.5 | 0.5 | 1 | -1 | 0.5 | -1 | -1 | -0.5 | -1 | -1 | -0.5 | 1 |
| KIRC | -1 | 1 | -1 | 1 | -1 | 0.5 | -1 | -1 | -1 | 0.5 | -1 | 1 |
| LIHC | 0.5 | 0.5 | 0.5 | -1 | 0.5 | -0.5 | -1 | 0.5 | 0.5 | -1 | 1 | 0.5 |
| LUAD | -0.5 | 0.5 | 1 | -1 | 0.5 | -1 | -1 | -0.5 | -1 | -1 | -0.5 | 1 |
| LUSC | 1 | 1 | 1 | -1 | -1 | -1 | -1 | 0.5 | 0.5 | -1 | -0.5 | 0.5 |
| PAAD | -0.5 | -0.5 | 0.5 | 0.5 | -0.5 | -0.5 | 0.5 | -0.5 | -0.5 | -0.5 | -0.5 | -0.5 |
| PRAD | 0.5 | 0.5 | 0.5 | -1 | 0.5 | 1 | -1 | 0.5 | 0.5 | -1 | 0.5 | -0.5 |
| STAD | 0.5 | 0.5 | 0.5 | -1 | -1 | -0.5 | -1 | 1 | -1 | -0.5 | -0.5 | 0.5 |
| Note. 1 was for significant overexpression and -1 was for significant underexpression in cancerous tissues, respectively. | | | | | | | | | | | | |
| 0.5 was for tentative overexpression and -0.5 was for tentative underexpression in cancerous tissues, respectively. | | | | | | | | | | | | |
